# *Ligusticum wallichii* Extract Inhibited the Expression of IL-1*β* after AMI in Rats

**DOI:** 10.1155/2014/620359

**Published:** 2014-08-17

**Authors:** Zhuo Yuan, Junping Zhang, Cui Yang

**Affiliations:** ^1^First Teaching Hospital of Tianjin University of Traditional Chinese Medicine, 314 An Shan Xi Road, Nan Kai District, Tianjin 300193, China; ^2^Tianjin University of Traditional Chinese Medicine, 312 An Shan Xi Road, Nan Kai District, Tianjin 300193, China

## Abstract

This study investigated the effects of *Ligusticum wallichii* on IL-1*β* expression in myocardium and central nervous system after AMI. AMI rat was administrated with *Ligusticum wallichii* extract. A series of assays were used to detect the effects of *Ligusticum wallichii* extract on infarct size, left ventricular ejection fraction, expression of TLR-4, NF-*κ*B, and IL-1*β* in myocardium, IL-1*β* expression in serum and hypothalamus, and NPY expression in hypothalamus. We observed that *Ligusticum wallichii* extract improved the left ventricular ejection fraction and reduced infarct area enlargement after AMI, by inhibiting the expression of IL-1*β* in myocardium, serum, and hypothalamus. *Ligusticum wallichii* extract reduced the expression of IL-1*β* in myocardium by regulating TLR4-NF-*κ*B signaling pathway and inhibited IL-1*β* in hypothalamus by regulating NPY mRNA expression.

## 1. Introduction

Myocardial infarction (MI) is a major cause of death globally; worldwide studies have indicated that MI is characterized by an intense inflammatory response within the myocardium. IL-1*β* is considered a key inflammatory mediator after acute myocardial infarction. IL-1*β* has been demonstrated to be significantly related to infarction and left ventricular function after MI [1, 2], and inhibited IL-1*β* expression could prevent heart failure after MI [3].

There are substantial evidences implicating that central nervous system- (CNS-) mediated mechanism involved the regulation of cardiac function after MI. Recent studies show that, following myocardial infarction elicited by coronary artery occlusion, there is an increase in IL-1*β* levels in the hypothalamus within 24 h after myocardial infarct. It has also elevated at the time when heart failure is established, approximately 6–8 weeks after a myocardial infarct in the rat [4]. The IL-1*β* elevated both in the circulation and in the hypothalamus after MI, but the relationship between them is not clear.

*Ligusticum wallichii* (ChuanXiong) is a Chinese medicinal herb that has been used orally with other herbs for heart [5] and brain diseases [6, 7] for thousands of years. Previous studies have indicated the possible link between the plant and heart disease.* L. wallichii* could improve blood fluidity [8] and inhibits endothelial cell damage [9] and vascular smooth muscle cell proliferation [10]. Current research shows that proinflammatory cytokines are modulators of cardiovascular function by a variety of mechanisms.* L. wallichii* was found to be related to anti-inflammatory activity by reducing serum TNF-*α*, IL-6 and IL-8 levels [11].* L. wallichii* also inhibited production of TNF-*α* and IL-1*β* activated by cerebral ischemia [12, 13].

An emerging area in cardiovascular research is the apparent significance of inflammation mechanisms. Many recent studies have suggested that anti-inflammation radicals may be important participants in a wide array of cardiac conditions, and several clinical trials evaluating the use of anti-inflammation as therapeutics either have already been conducted or are underway. The present study was designed to investigate the effects of* Ligusticum wallichii* extract on anti-inflammation activities in AMI rats.

## 2. Materials and Methods

### 2.1. Materials

The reagents used in the study were purchased as follows: pentobarbital sodium from Sigma, America; ELISA kits for IL-1*β* from Jiancheng Biotech, Nanjing, China; NBT from Light Biotech, Beijing, China; mice anti-rat toll-like receptor 4 (TLR4) and mice anti-rat TNF receptor associated with factor-6 (TRAF-6) from Boster Biotech, Wuhan, China; mice anti-rat NF-*κ*B p65 from Santa Cruz, America; IL-1*β* monoclonal antibodies from Sigma, America; SP kits and DAB kits from ZhongShanJinqiao Biotech, Beijing, China; prestained protein ladder 10–170 kDa from Thermo Scientific, America;* Ligusticum wallichii* from First Teaching Hospital of Tianjin university of TCM, lot number Y20050124; aspirin from Bayer, National License Medical Number J20080078.

### 2.2. Preparation of* Ligusticum wallichii* Extract

A batch of 63 g of the* Ligusticum wallichii* was soaked in 1000 mL deionized water for 0.5 h and continuously extracted using deionized water at boiling point for 1 h; the extracts were collected. The herbs were then soaked in 800 mL deionized water extracted for another 1 h. Then, all these extracts were mixed, filtered, concentrated, dried, and weighed.

### 2.3. Animals, AMI Induction, and Treatment

Sprague-Dawley rats (180 ± 50 g, 8–10 weeks) were obtained from the experimental animal center of Military Medical Science Academy, Tianjin, China. They were housed in groups (4 per cage) with free access to a regular diet and clean drinking water. All the experimental procedures described below adhered strictly to the guidelines set forth by the National Science and Technology Commission of China and approved by the institutional ethics committee.

For AMI induction, rats were anesthetized with an intraperitoneal dose of 0.2 mL 2% pentobarbital under intubated mechanical ventilation at 80 breaths per minute. Thoracotomy was performed through the fourth intercostal space and the left anterior descending coronary artery was identified and ligated with 6.0 prolene suture in the middle portion. A few minutes after ligation, pallor and akinesia were seen in the anterior wall and apical left ventricular area. The interface between the pale and normal areas was defined as “infarction border zone.” The presence of infarction zone and ST segment elevation were considered as criteria of successful induction of AMI. A group of animals (*n* = 10) were left untreated controls (sham group). The animals that were successfully induced to develop AMI were randomized to AMI group and received PBS (10 mg/Kg/d, *n* = 10),* Ligusticum wallichii* group (10 mg/Kg/d, *n* = 10) and aspirin group (10 mg/Kg/d, *n* = 10), respectively.

### 2.4. Left Ventricular Ejection Fraction Evaluation

Echocardiographic examinations were performed 7 days after surgery, with the Philips Sonos 5500 ultrasound system (HP, Germany). The frequency of the probe was set at 17.5 MHz, sampling frequency in M-mode at 1000/s, and scanning speed 50–100 mm/s. The probes were placed on precordium and the detection was carried out from the section of ventricular bands. Left ventricular anterior wall (LVAW) thickness, left ventricular end-diastolic diameters (LVEDD), and left ventricular end-systolic diameters (LVESD) were measured. Based on these measurements, left ventricular end-diastolic volume (LVEDV), left ventricular end-systolic volume (LVESV), and left ventricular ejection fraction (LVEF) were calculated as follows:

(1) $$\begin{array}{c} \text{LVEDV}=\frac{7.0\times {\text{LVEDD}}^{3}}{\left(2.4+\text{LVEDD}\right)},\\ \text{LVESV}=\frac{7.0\times {\text{LVESD}}^{3}}{\left(2.4+\text{LVESD}\right)},\\ \text{LVEF}=\frac{\text{LVEDV}-\text{LVESV}}{\text{LVEDV}}\times 100\%. \end{array}$$

### 2.5. Infarct Area Size Assessment

Areas identified as infarct, nonischemic based on NBT staining were measured by computerized video planimetry and from these measurements infarct size (MIS) was calculated as a percentage of the region at risk [14]. MIS = ischemia area weight/ventricular weight × 100%.

### 2.6. Expression of TLR4, TRAF-6, NF-*κ*B, and IL-1*β* in Myocardium by Immunohistochemical Staining

Sections were deparaffinized by a standard method, cut, fixed in 100% acetone, and stored at −20°C. The mouse anti-rat TLR4, TNF receptor associated to factor-6 (TRAF-6), NF-*κ*B and IL-1*β* polyclonal antibody were used for the identification of TLR4, TRAF-6, NF-*κ*B, and IL-1*β* positive cells. Sections were washed twice in Tris HCl and pH 7.6 and briefly in buffer containing 1% polymerized bovine albumin.

The sections were incubated for 1 hour at room temperature with the primary antibodies diluted 1 : 100 for TLR4, 1 : 150 for TRAF-6, 1 : 25 for NF-*κ*B, and 1 : 200 for IL-1*β*. And then the secondary antibody is biotinylated and the label is peroxidase conjugated streptavidin. Control sections were treated with the same procedure except they were incubated without the specific primary antibodies. And then diaminobenzidine (DAB) staining was performed. Positive signals were quantified using the Image Pro-Plus 5.1 software. The number of cells positive for TLR4, TRAF-6, NF-*κ*B, or IL-1*β* was counted in 10 high power fields (×400).

### 2.7. Measurement of IL-1*β* in Serum by ELISA

The cytokine measurements were performed according to the manufacturer's instructions for the ELISA kit (BD Biosciences, USA).

### 2.8. Measurement of IL-1*β* and NPY in Myocardium and Hypothalamus by Real-Time Quantitative RT-PCR Analysis

Total RNA was extracted using a commercial RNAprep pure kit (number DP430, Tiangen, China). Total RNA concentration was determined from spectrophotometric optical density measurement (260 and 280 nm). For each sample tested, the ratio between the spectrophotometric readings at 260 nm and 280 nm (OD260/OD280) was used to provide an estimate of the purity of the nucleic acid, and the ratio in all samples ranged between 1.8 and 2.0.

Total RNA was reverse transcribed at 46°C for 60 min, 70°C for 5 min, and 5°C for 5 min using the PrimeScript RT master mix kit (number DRR036, TaKaRa, Japan) and then cDNA was stored at −20°C. Quantitative PCR reactions were carried out with the SYBR Premix Ex TaqTM II kit (number DRR081, TaKaRa, Japan) in a thermocycler (Applied Biosystems 7500 Real-Time PCR System, USA). Amplification conditions included 95°C for 10 s followed by 39 cycles of 95°C for 5 s and 60°C for 20 s. The forward IL-1*β* primer was 5′-TGTGATGTTCCCATTAGAC-3′, and the reverse IL-1*β* primer was 5′-AATACCACTTGTTGGCTTA-3′ (131 bp product). The forward GAPDHP primer was 5′-CTGATGCCTCCATGTTTGTG-3′; the reverse GAPDHP was 5′-GGATGCAGGGATGATGTTCT-3′ (254 bp product). The forward NPY primer was 5′-CTGACCCTCGCTCTATCC-3′, and the reverse NPY primer was 5′-GGTCTTCAAGCCTTGTTCT-3′ (247 bp product). The forward GAPDHP primer was 5′-CTGATGCCTCCATGTTTGTG-3′; the reverse GAPDHP was 5′-GGATGCAGGGATGATGTTCT-3′ (254 bp product).

Amplification specificity was verified by the melting curve following the manufacturer's instructions and 1.5% agarose gel electrophoresis. The data were normalized to the Ct value of the internal housekeeping gene beta-actin and the relative mRNA level in the untreated group was used as calibrator. Fold change of the target gene mRNA expression was calculated using the 2-ΔΔCT method.

### 2.9. Statistical Analysis

Values of all experiments were represented as mean ± SD. Statistical significance was assessed by one-way ANOVA followed by LSD post hoc multiple comparisons. The level of significance was set at *P* < 0.05.

## 3. Result

### 3.1. *Ligusticum wallichii* Extract Improved the Left Ventricular Ejection Fraction (LVEF)

**Figure 1:**
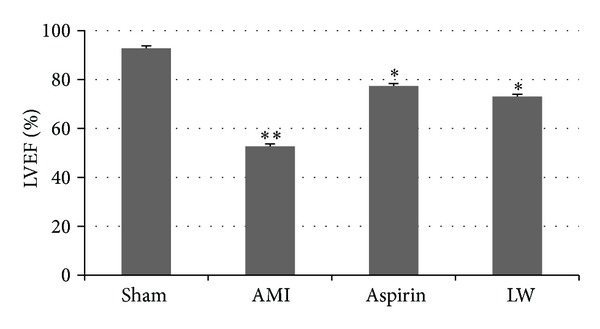
Left ventricular ejection fraction was assessed by echocardiograph. The LVEF was significantly decreased in the AMI group when compared with the sham group (**P < 0.01).* Ligusticum wallichii* (10 mg/Kg/d body weight) and aspirin (10 mg/Kg/d body weight) could improve the LVEF of AMI rat versus AMI group (*P < 0.05). But there were no remarkable differences between LW group and aspirin group.

The left ventricular ejection fraction was assessed by echocardiograph (Figure 1). The LVEF was significantly decreased in the AMI group when compared with the sham group (*P* < 0.01). However, that alternation was reduced (*P* < 0.05) by feeding LW and aspirin. But there were no remarkable differences between LW group and aspirin group.

### 3.2. *Ligusticum wallichii* Extract Reduced Infarct Area Size

**Figure 2:**
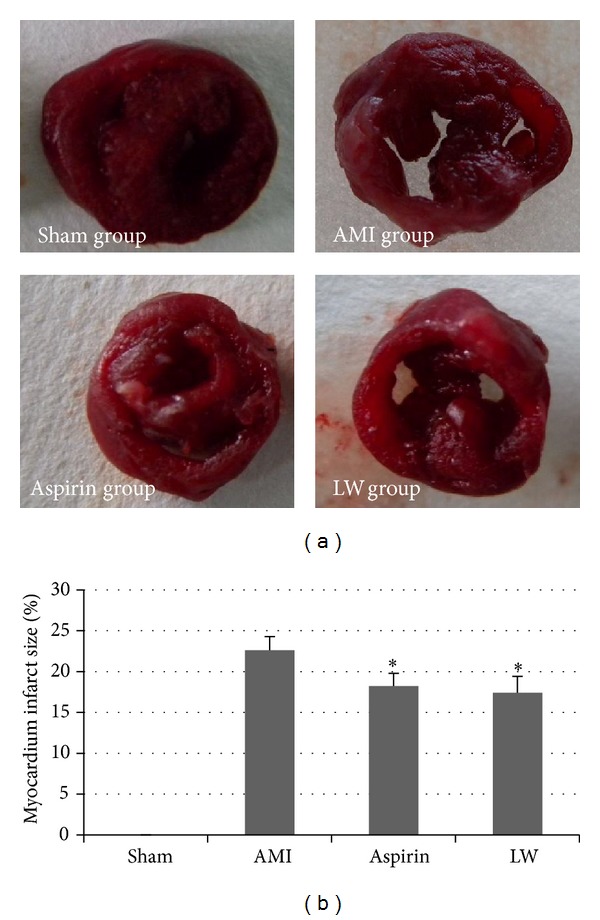
Representative images for infarcted hearts at one week time point after AMI. (a) NBT staining of infarcted area. The color of myocardium in the control group was dark blue, but the infarct area was hoar. (b)* Ligusticum wallichii* (10 mg/Kg/d body weight) and aspirin (10 mg/Kg/d body weight) reduced the MIS versus AMI group (*P < 0.05). But there were no remarkable differences between LW group and aspirin group.

The infarct area was assessed by NBT staining (Figure 2). NBT stain showed that the color of myocardium in the control group was dark blue, but the infarct area of AMI model group was hoar. One week after infarction, a large infarcted area with a collapsed and pale left ventricular wall was seen under the ligated silk. Compared to the sham group, larger infarcted area was detected accompanying global enlargement of the heart in the AMI group.* Ligusticum wallichii* and aspirin treatment reduced infarct area enlargement.

### 3.3. *Ligusticum wallichii* Extract Inhibited the Expression of IL-1*β* in Myocardium, Serum, and Hypothalamus

**Figure 3:**
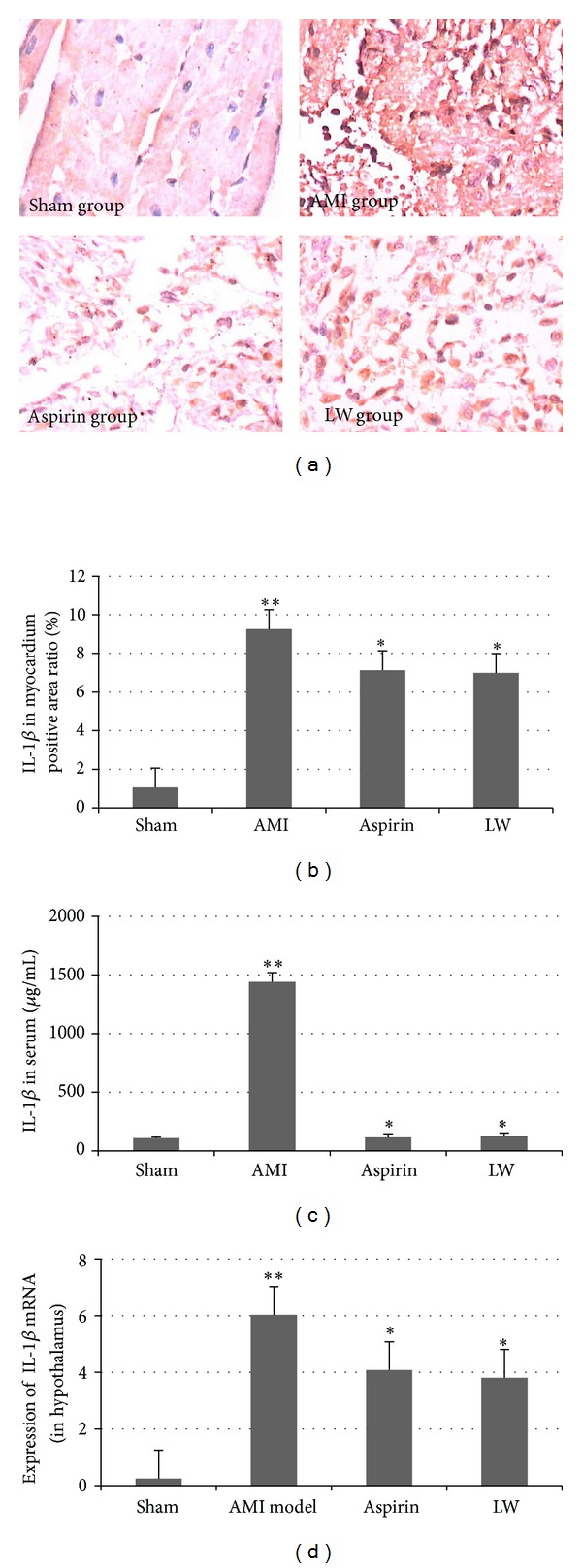
*Ligusticum wallichii* extract inhibited the expression of IL-1*β*. (a) Representative micrographs were taken at a magnification of ×400. The brown areas were IL-1*β* positive cell; the brown areas were significantly increased in the AMI model group.* Ligusticum wallichii* and aspirin decreased the brown area. (b) In the sham group, the immunoreactive staining occurred less in the cytoplasm. The number of IL-1*β*-like immunoreactive cells increased significantly in the myocardium after AMI when compared with the sham group (**P < 0.01).* Ligusticum wallichii* (10 mg/Kg/d body weight) and aspirin (10 mg/Kg/d body weight) both inhibited IL-1*β* protein expression versus AMI group (*P < 0.05), but there were no remarkable differences between LW group and aspirin group. (c) Serum IL-1*β* level was assessed by ELISA kit. The serum IL-1*β* level was significantly upregulated 1w after AMI when compared with the sham group (**P < 0.01).* Ligusticum wallichii* (10 mg/Kg/d body weight) and aspirin (10 mg/Kg/d body weight) both inhibited the IL-1*β* overexpression after AMI versus AMI model (*P < 0.05), but there were no remarkable differences between LW group and aspirin group. (d) Effect of* Ligusticum wallichii* on the IL-1*β* mRNA expression in the hypothalamus of AMI rats. Quantitative RT-PCR was performed. The IL-1*β* mRNA expression was significantly upregulated 1w after AMI versus sham group (**P < 0.01).* Ligusticum wallichii* and aspirin inhibited the hypothalamus IL-1*β* mRNA expression versus AMI group (*P < 0.05), but there were no remarkable differences between LW group and aspirin group.

IL-1*β* immunohistochemical staining could be detected to the cells of rat myocardium after AMI (Figures 3(a) and 3(b)). The IL-1*β* protein was predominantly expressed in the cytoplasm. Few cells positive for IL-1*β*-like immunoreaction were observed in the sham group. Intensive IL-1*β*-like immunostaining was present in myocardium after AMI. Significant changes of IL-1*β* protein expression were observed in the LW group and aspirin group.

The serum IL-1*β* level was assessed by ELISA kit (Figure 3(c)). The serum IL-1*β* level was elevated 1W after AMI. LW and aspirin inhibited the IL-1*β* overexpression in serum, but there were no remarkable differences between LW group and aspirin group.

The hypothalamus IL-1*β* mRNA expression was assessed by quantitative reverse transcription-PCR (Figure 3(d)). The hypothalamus IL-1*β* mRNA expression was elevated 1W after AMI. LW and aspirin inhibited IL-1*β* mRNA expression in hypothalamus, but there were no remarkable differences between LW group and aspirin group.

### 3.4. *Ligusticum wallichii* Extract Inhibited the Expression of TLR4, TRAF-6, and NF-*κ*B in Myocardium

**Figure 4:**
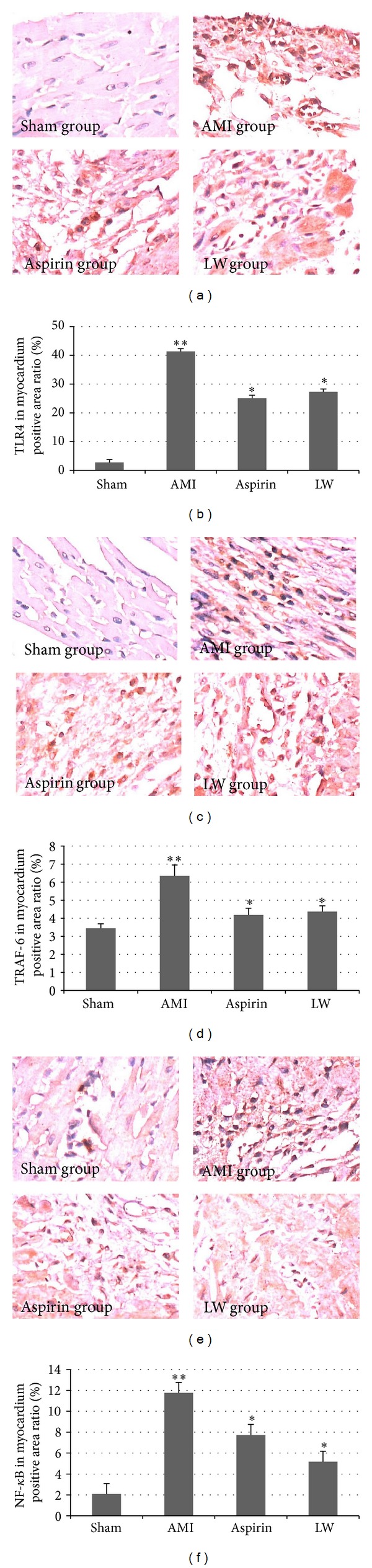
*Ligusticum wallichii* extract inhibited the expression of TLR4, TRAF-6, NF-*κ*B in myocardium. (a) Representative micrographs were taken at a magnification of ×400. The brown areas were TLR4 positive cell; the brown areas were significantly increased in the AMI model group.* Ligusticum wallichii* and aspirin decreased the brown area. (b) In the sham group, the immunoreactive staining occurred less in the cytoplasm. The number of TLR4-like immunoreactive cells increased significantly in the myocardium after AMI when compared with the sham group (**P < 0.01).* Ligusticum wallichii* (10 mg/Kg/d body weight) and aspirin (10 mg/Kg/d body weight) both inhibited TLR4 protein expression versus AMI group (*P < 0.05), but there were no remarkable differences between LW group and aspirin group. (c) The brown areas were TRAF-6 positive cell; the brown areas were significantly increased in the AMI model group.* Ligusticum wallichii* and aspirin decreased the brown area. (d) In the sham group, the immunoreactive staining occurred less in the cytoplasm. The number of TRAF-6-like immunoreactive cells increased significantly in the myocardium after AMI when compared with the sham group (**P < 0.01).* Ligusticum wallichii* and aspirin both inhibited TRAF-6 protein expression versus AMI group (*P < 0.05), but there were no remarkable differences between LW group and aspirin group. (e) The brown areas were NF-*κ*B positive cell; the brown areas were significantly increased in the AMI model group.* Ligusticum wallichii* and aspirin decreased the brown area. (f) In the sham group, the immunoreactive staining occurred less in the cytoplasm. The number of NF-*κ*B-like immunoreactive cells increased significantly in the cytoplasm and nucleus after AMI.* Ligusticum wallichii* and aspirin both inhibited NF-*κ*B protein expression versus AMI group (*P < 0.05).

TLR4 (Figures 4(a) and 4(b)) and TRAF-6 (Figures 4(c), 4(d)) immunohistochemical staining could be detected to the cells of rat myocardium after AMI. The TLR4 and TRAF-6 protein was predominantly expressed in the cytoplasm. Few cells positive for TLR4-like and immunoreaction were observed in the sham group. Intensive TLR4-like and TRAF-6-like immunostaining was present in myocardium after AMI. Significant changes of TLR4 and TRAF-6 protein expression were observed in the* Ligusticum wallichii* group and aspirin group.

NF-*κ*B immunohistochemical staining could be detected to the cells of rat myocardium after AMI (Figures 4(e) and 4(f)). In sham group, the NF-*κ*B protein was predominantly expressed in the cytoplasm. However, after AMI, expression was observed both in the cytoplasm and nucleus. Few cells positive for NF-*κ*B-like immunoreaction were observed in the sham group. Intensive NF-*κ*B-like immunostaining was present in myocardium after AMI. Significant changes of NF-*κ*B protein expression were observed in the* Ligusticum wallichii* group and aspirin group.

### 3.5. *Ligusticum wallichii* Extract Inhibited NPY mRNA Expression in Hypothalamus

**Figure 5:**
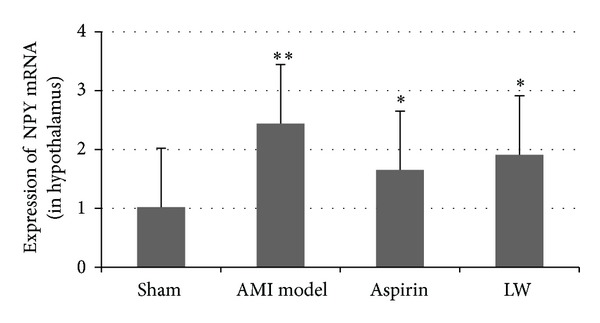
Effect of* Ligusticum wallichii* on the NPY mRNA expression in the hypothalamus of AMI rats. Quantitative RT-PCR was performed. The NPY mRNA expression was significantly upregulated 1w after AMI versus sham group (**P < 0.01).* Ligusticum wallichii* and aspirin inhibited the hypothalamus NPY mRNA expression versus AMI group (*P < 0.05), but there were no remarkable differences between LW group and aspirin group.

The hypothalamus NPY mRNA expression was assessed by quantitative reverse transcription-PCR (Figure 5). The hypothalamus NPY mRNA expression was elevated 1W after AMI. LW and aspirin inhibited NPY mRNA expression in hypothalamus, but there were no remarkable differences between LW group and aspirin group.

## 4. Discussion

The objective of our study was to investigate the effects of* Ligusticum wallichii* extract on anti-inflammation activities after AMI. We found that* Ligusticum wallichii* extract (10 mg/Kg/d body weight) could improve the left ventricular ejection fraction and reduced infarct area enlargement.* Ligusticum wallichii* extract also inhibited the expression of IL-1*β* in myocardium, serum, and hypothalamus. Previous studies have indicated that MI is characterized by an intense inflammatory response. IL-1*β* is considered a key inflammatory mediator after acute myocardial infarction. IL-1*β* has been demonstrated to be significantly related to infarction and left ventricular function after MI [2]. Recent studies show that, following myocardial infarction elicited by coronary artery occlusion, there is an increase in IL-1*β* levels in the hypothalamus within 24 h after myocardial infarct [15]. And inhibited brain IL-1*β* synthesis could reduce infarction and improve left ventricular function [16], this suggests that inhibiting IL-1*β* expression in brain could improve heart function.

Our results identify that* Ligusticum wallichii* extract (10 mg/Kg/d body weight) could inhibit the expression of TLR4 and NF-*κ*B in myocardium after AMI. Toll-like receptors (TLRs) play an important role in the regulation of innate immune and inflammatory responses by recognition of pathogen associated molecular patterns (PAMPs) that are not present in the host [17]. TLR4 is a member of the TLRs that have natural pattern recognition. The function of TLR4 is to mediate transmembrane signaling transduction in which TLR4 could serve as a bridge that links innate immunity and vascular inflammation. TLR4 widely recognizes specific pathogen-associated molecular patterns, such as couples signal transduction pathways to activate inflammatory cells, which results in a series of inflammatory responses and leads to the synthesis and release of cytokines and inflammatory mediators. In TLR4-deficient mice, this vascular proinflammatory gene cannot be expressed, regardless of the extent of obesity, dyslipidemia, or high fat intake [18].

Nuclear factor (NF)-*κ*B is downstream of the signaling pathway activating IL-1*β*; NF-*κ*B pathway plays an important role in TLR4-mediated inflammatory regulation [19]. It is an essential transcription factor that regulates inflammatory responses through modulation of the expression of various proinflammatory mediators, including cytokines and NO. NF-*κ*B is also a primary regulator of genes that are involved in the production of proinflammatory cytokines and enzymes involved in the process of inflammation [20, 21]. TLR4 was upregulated in response to IL-1*β*. IL-1*β* activates NF-*κ*B resulting in transcriptional activation of a wide variety of genes such as inflammatory mediators.

Tumor necrosis factor (TNF) receptor associated factors (TRAFs) play important roles in intracellular signal transduction of many receptor families such as the IL-1 receptors (IL-1R) [22]. They could lead to activation of transcription factors such as NF-*κ*B and inflammatory responses. Remarkably, TRAF6 is uniquely pleiotropic in participating in the signal transduction of many receptor systems while TRAF2, TRAF3, and TRAF5 appear to signal only within the TNF receptor superfamily [23]. TRAF-6 could be active by TLR4 and then activate the inhibitor of *κ*B (I*κ*B) kinase (IKK), leading ultimately to activation of NF-*κ*B [24]. Levels of TRAF-6 are related to inflammation in CAD patients [25].

Neuropeptide Y (NPY) is one of the most abundant neuropeptides present in the human peripheral and central nervous systems [26]. It acts as a neurotransmitter, regulating various autonomic and endocrine functions [27]. Specifically, NPY-containing neurons are present in the paraventricular nucleus (PVN) of the hypothalamus, the ventrolateral medulla (VLM), the NTS, and the sympathetic fibres that innervate blood vessels [28]. NPY played a significant role in central cardiovascular regulation [29, 30]. More recently, genome wide association studies have linked NPY to human coronary artery disease (CAD): SNPs in the NPY gene correlated to CAD in humans and even more so in early onset patients [31].

In recent years, NPY has been also described to play a pivotal role in the immune system; NPY can increase the IL-1*β* secretion [32]. And NPY receptors are present on the surface of various leukocyte subgroups modulating the release of different cytokines. NPY Y1 receptor signaling could prevent NF-*κ*B activation triggered by IL-1*β* [33]. In our study,* Ligusticum wallichii* extract inhibited the expression of NPY in hypothalamus, suggesting that it could reduce the IL-1*β* level in hypothalamus after AMI by inhibiting NPY mRNA expression.

## 5. Conclusion

*Ligusticum wallichii* extract improved the left ventricular ejection fraction and reduced infarct area enlargement after AMI, by inhibiting the expression of IL-1*β* in myocardium, serum, and hypothalamus.* Ligusticum wallichii* extract reduced the expression of IL-1*β* in myocardium by regulating TLR4-NF-*κ*B signaling pathway and inhibited the expression of IL-1*β* in hypothalamus by regulating NPY mRNA expression.
